# 2-Chloro-*N*-(3,4-dimethyl­phen­yl)acetamide

**DOI:** 10.1107/S1600536809013051

**Published:** 2009-04-10

**Authors:** B. Thimme Gowda, Sabine Foro, Hiromitsu Terao, Hartmut Fuess

**Affiliations:** a Department of Chemistry, Mangalore University, Mangalagangotri 574 199, Mangalore, India; b Institute of Materials Science, Darmstadt University of Technology, Petersenstrasse 23, D-64287 Darmstadt, Germany; c Faculty of Integrated Arts and Sciences, Tokushima University, Minamijosanjima-cho, Tokushima 770-8502, Japan

## Abstract

The conformation of the C=O bond in the structure of the title compound, C_10_H_12_ClNO, is *anti* to the N—H bond and to the methyl­ene H atoms in the side chain in both the independent mol­ecules comprising the asymmetric unit. However, the conformation of the N—H bond is *syn* to the *meta*-methyl substituent in the aromatic ring of one of the mol­ecules and *anti* in the other mol­ecule. The two independent mol­ecules are linked through inter­molecular N—H⋯O hydrogen bonding into chains parallel to the *b* axis.

## Related literature

For preparation of the compound, see: Shilpa & Gowda (2007 ▶). For related structures, see: Gowda *et al.* (2008*a* ▶,*b* ▶,*c* ▶).
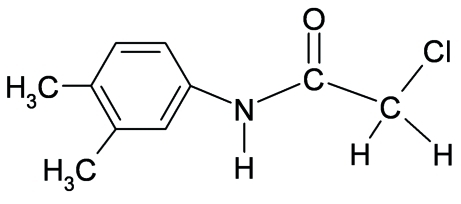


## Experimental

### 

#### Crystal data

C_10_H_12_ClNO
*M* *_r_* = 197.66
Triclinic, 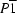

*a* = 8.3672 (9) Å
*b* = 9.8076 (9) Å
*c* = 12.409 (1) Å
α = 95.415 (8)°
β = 96.492 (9)°
γ = 97.767 (9)°
*V* = 996.26 (16) Å^3^
*Z* = 4
Mo *K*α radiation
μ = 0.34 mm^−1^
*T* = 299 K
0.44 × 0.36 × 0.20 mm

#### Data collection

Oxford Diffraction Xcalibur diffractometer with a Sapphire CCD detector
Absorption correction: multi-scan (*CrysAlis RED*; Oxford Diffraction, 2007 ▶) *T*_min_ = 0.868, *T*_max_ = 0.936
10754 measured reflections
3634 independent reflections
2874 reflections with *I* > 2σ(*I*)
*R*_int_ = 0.017

#### Refinement

*R*[*F* ^2^ > 2σ(*F* ^2^)] = 0.035
*wR*(*F* ^2^) = 0.127
*S* = 0.97
3634 reflections
240 parameters
H-atom parameters constrained
Δρ_max_ = 0.19 e Å^−3^
Δρ_min_ = −0.23 e Å^−3^

### 

Data collection: *CrysAlis CCD* (Oxford Diffraction, 2004 ▶); cell refinement: *CrysAlis RED* (Oxford Diffraction, 2007 ▶); data reduction: *CrysAlis RED*; program(s) used to solve structure: *SHELXS97* (Sheldrick, 2008 ▶); program(s) used to refine structure: *SHELXL97* (Sheldrick, 2008 ▶); molecular graphics: *PLATON* (Spek, 2009 ▶); software used to prepare material for publication: *SHELXL97*.

## Supplementary Material

Crystal structure: contains datablocks I, global. DOI: 10.1107/S1600536809013051/dn2441sup1.cif

Structure factors: contains datablocks I. DOI: 10.1107/S1600536809013051/dn2441Isup2.hkl

Additional supplementary materials:  crystallographic information; 3D view; checkCIF report

## Figures and Tables

**Table 1:** Hydrogen-bond geometry (Å, °)

*D*—H⋯*A*	*D*—H	H⋯*A*	*D*⋯*A*	*D*—H⋯*A*
N1—H1*N*⋯O2	0.86	2.05	2.8992 (16)	172
N2—H2*N*⋯O1^i^	0.86	2.14	2.9802 (16)	166

Symmetry code: (i) 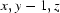.

**Fig. 1.**
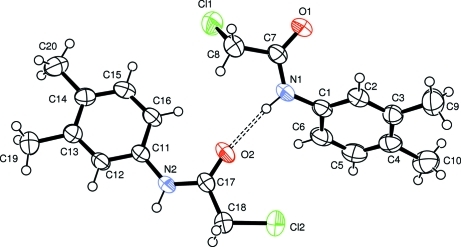
Molecular structure of (I), showing the atom labeling scheme and displacement ellipsoids drawn at the 50% probability level.

**Fig. 2.**
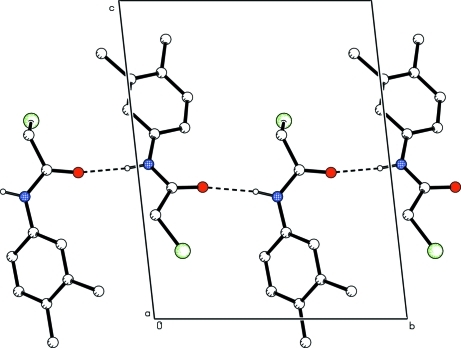
Molecular packing of (I) with hydrogen bonding shown as dashed lines.

## supplementary crystallographic information

### Comment

In the present work, as part of a study of the effect of ring and side chain substitutions on the crystal structures of aromatic amides (Gowda *et al.*, 2008a,b,c), the structure of 2-chloro-*N*-(3,4-dimethylphenyl)acetamide (I) has been determined. The asymmetric unit of the structure contains two molecules (Fig. 1). The conformation of the N—H bond is *syn* to the *meta*-methyl substituent in the aromatic ring of one of the molecules and *anti* in the other molecule. The conformation of the C=O bond in the structure is *anti* to the N—H bond and to the side chain methylene H-atoms in the side chain (Fig. 1), in both the independent molecules comprising the asymmetric unit, similar to that observed in in 2-chloro-*N*- (2,4-dimethylphenyl)acetamide (Gowda *et al.*, 2008*a*), in 2-chloro-*N*-(3,5-dichlorophenyl)acetamide (Gowda *et al.*, 2008*b*), and in 2-chloro-*N*-(3-methylphenyl)acetamide (Gowda *et al.*, 2008*c*). The two independent molecules in (I) are linked through intermolecular N—H···O hydrogen bonding (Fig.1) and the chains formed by H-bonding are parallel to the *b* axis (Table 1, Fig. 2).

### Experimental

Compound (I) was prepared according to the literature method (Shilpa & Gowda, 2007). Single crystals were obtained from the slow evaporation of an ethanolic solution of (I).

### Refinement

The H atoms were positioned with idealized geometry using a riding model with C—H = 0.93–0.97 Å, N—H = 0.86 Å, and were refined with isotropic displacement parameters (set to 1.2 times of the *U*_eq_ of the parent atom).

### Crystal data

**Table:**

C_10_H_12_ClNO	*Z* = 4
*M**_r_* = 197.66	*F*(000) = 416
Triclinic, *P*1	*D*_x_ = 1.318 Mg m^−^^3^
Hall symbol: -P 1	Mo *K*α radiation, λ = 0.71073 Å
*a* = 8.3672 (9) Å	Cell parameters from 4345 reflections
*b* = 9.8076 (9) Å	θ = 2.8–27.6°
*c* = 12.409 (1) Å	µ = 0.34 mm^−^^1^
α = 95.415 (8)°	*T* = 299 K
β = 96.492 (9)°	Rod, colourless
γ = 97.767 (9)°	0.44 × 0.36 × 0.20 mm
*V* = 996.26 (16) Å^3^	

### Data collection

**Table:**

Oxford Diffraction Xcalibur diffractometer with a Sapphire CCD detector	3634 independent reflections
Radiation source: fine-focus sealed tube	2874 reflections with *I* > 2σ(*I*)
graphite	*R*_int_ = 0.017
Rotation method data acquisition using ω and φ scans	θ_max_ = 25.4°, θ_min_ = 2.8°
Absorption correction: multi-scan (*CrysAlis RED*; Oxford Diffraction, 2007)	*h* = −10→10
*T*_min_ = 0.868, *T*_max_ = 0.936	*k* = −11→11
10754 measured reflections	*l* = −14→14

### Refinement

**Table:**

Refinement on *F*^2^	Secondary atom site location: difference Fourier map
Least-squares matrix: full	Hydrogen site location: inferred from neighbouring sites
*R*[*F*^2^ > 2σ(*F*^2^)] = 0.035	H-atom parameters constrained
*wR*(*F*^2^) = 0.127	*w* = 1/[σ^2^(*F*_o_^2^) + (0.1*P*)^2^] where *P* = (*F*_o_^2^ + 2*F*_c_^2^)/3
*S* = 0.97	(Δ/σ)_max_ < 0.001
3634 reflections	Δρ_max_ = 0.19 e Å^−^^3^
240 parameters	Δρ_min_ = −0.23 e Å^−^^3^
0 restraints	Extinction correction: *SHELXL97* (Sheldrick, 2008), Fc^*^=kFc[1+0.001xFc^2^λ^3^/sin(2θ)]^-1/4^
Primary atom site location: structure-invariant direct methods	Extinction coefficient: 0.024 (4)

### Special details

**Table:**

Experimental. Absorption correction: empirical absorption correction using spherical harmonics, implemented in SCALE3 ABSPACK scaling algorithm, CrysAlis RED (Oxford Diffraction, 2007).
Geometry. All e.s.d.'s (except the e.s.d. in the dihedral angle between two l.s. planes) are estimated using the full covariance matrix. The cell e.s.d.'s are taken into account individually in the estimation of e.s.d.'s in distances, angles and torsion angles; correlations between e.s.d.'s in cell parameters are only used when they are defined by crystal symmetry. An approximate (isotropic) treatment of cell e.s.d.'s is used for estimating e.s.d.'s involving l.s. planes.
Refinement. Refinement of *F*^2^ against ALL reflections. The weighted *R*-factor *wR* and goodness of fit *S* are based on *F*^2^, conventional *R*-factors *R* are based on *F*, with *F* set to zero for negative *F*^2^. The threshold expression of *F*^2^ > σ(*F*^2^) is used only for calculating *R*-factors(gt) *etc*. and is not relevant to the choice of reflections for refinement. *R*-factors based on *F*^2^ are statistically about twice as large as those based on *F*, and *R*- factors based on ALL data will be even larger.

### Fractional atomic coordinates and isotropic or equivalent isotropic displacement parameters (Å^2^)

**Table:**

	*x*	*y*	*z*	*U*_iso_*/*U*_eq_	
Cl1	0.62739 (6)	0.60024 (5)	0.62240 (4)	0.0701 (2)	
O1	0.83974 (16)	0.76355 (11)	0.45971 (10)	0.0569 (3)	
N1	0.81699 (17)	0.54085 (13)	0.38380 (11)	0.0477 (3)	
H1N	0.8113	0.4570	0.3998	0.057*	
C1	0.81633 (19)	0.55916 (15)	0.27115 (14)	0.0447 (4)	
C2	0.9174 (2)	0.66380 (16)	0.23435 (14)	0.0482 (4)	
H2	0.9875	0.7272	0.2846	0.058*	
C3	0.9155 (2)	0.67553 (17)	0.12342 (14)	0.0490 (4)	
C4	0.8120 (2)	0.57967 (18)	0.04769 (14)	0.0542 (4)	
C5	0.7129 (2)	0.47505 (18)	0.08636 (16)	0.0627 (5)	
H5	0.6434	0.4105	0.0367	0.075*	
C6	0.7148 (2)	0.46423 (17)	0.19551 (16)	0.0575 (5)	
H6	0.6474	0.3926	0.2190	0.069*	
C7	0.82545 (19)	0.63949 (16)	0.46785 (14)	0.0446 (4)	
C8	0.8184 (2)	0.58415 (19)	0.57750 (14)	0.0546 (4)	
H8A	0.9049	0.6356	0.6304	0.066*	
H8B	0.8339	0.4876	0.5710	0.066*	
C9	1.0278 (3)	0.7913 (2)	0.08682 (19)	0.0753 (6)	
H9A	1.0907	0.8458	0.1495	0.090*	
H9B	0.9646	0.8488	0.0466	0.090*	
H9C	1.0995	0.7528	0.0411	0.090*	
C10	0.8062 (3)	0.5877 (3)	−0.07390 (16)	0.0781 (6)	
H10A	0.9126	0.5838	−0.0949	0.094*	
H10B	0.7710	0.6731	−0.0913	0.094*	
H10C	0.7314	0.5112	−0.1126	0.094*	
Cl2	0.90424 (8)	0.14166 (5)	0.21372 (4)	0.0779 (2)	
O2	0.78329 (17)	0.24929 (11)	0.41405 (11)	0.0628 (4)	
N2	0.74319 (15)	0.04521 (12)	0.48565 (10)	0.0414 (3)	
H2N	0.7539	−0.0406	0.4734	0.050*	
C11	0.67313 (18)	0.08227 (14)	0.58097 (12)	0.0379 (3)	
C12	0.66625 (19)	−0.01217 (15)	0.65716 (12)	0.0428 (4)	
H12	0.7076	−0.0946	0.6435	0.051*	
C13	0.5996 (2)	0.01258 (16)	0.75308 (13)	0.0458 (4)	
C14	0.53726 (19)	0.13642 (16)	0.77369 (13)	0.0459 (4)	
C15	0.5432 (2)	0.22884 (16)	0.69622 (14)	0.0503 (4)	
H15	0.5011	0.3110	0.7092	0.060*	
C16	0.60886 (19)	0.20418 (15)	0.60076 (14)	0.0466 (4)	
H16	0.6102	0.2683	0.5501	0.056*	
C17	0.79504 (19)	0.12730 (15)	0.41182 (13)	0.0425 (4)	
C18	0.8760 (2)	0.05019 (18)	0.32656 (13)	0.0519 (4)	
H18A	0.8097	−0.0386	0.3018	0.062*	
H18B	0.9808	0.0326	0.3598	0.062*	
C19	0.5979 (3)	−0.0922 (2)	0.83395 (16)	0.0698 (6)	
H19A	0.6609	−0.0513	0.9016	0.084*	
H19B	0.6439	−0.1707	0.8058	0.084*	
H19C	0.4879	−0.1216	0.8462	0.084*	
C20	0.4663 (2)	0.1688 (2)	0.87824 (15)	0.0639 (5)	
H20A	0.5468	0.1673	0.9396	0.077*	
H20B	0.3736	0.1008	0.8819	0.077*	
H20C	0.4333	0.2589	0.8797	0.077*	

### Atomic displacement parameters (Å^2^)

**Table:**

	*U*^11^	*U*^22^	*U*^33^	*U*^12^	*U*^13^	*U*^23^
Cl1	0.0802 (4)	0.0660 (3)	0.0721 (3)	0.0167 (3)	0.0302 (3)	0.0161 (2)
O1	0.0811 (9)	0.0320 (6)	0.0606 (7)	0.0144 (5)	0.0136 (6)	0.0069 (5)
N1	0.0598 (9)	0.0290 (6)	0.0568 (8)	0.0085 (6)	0.0128 (7)	0.0085 (6)
C1	0.0469 (9)	0.0323 (7)	0.0568 (9)	0.0115 (7)	0.0090 (7)	0.0041 (7)
C2	0.0470 (9)	0.0380 (8)	0.0578 (10)	0.0046 (7)	0.0043 (8)	0.0017 (7)
C3	0.0500 (10)	0.0447 (9)	0.0559 (10)	0.0142 (7)	0.0101 (8)	0.0097 (7)
C4	0.0570 (10)	0.0513 (10)	0.0570 (10)	0.0243 (8)	0.0029 (8)	0.0020 (8)
C5	0.0653 (12)	0.0478 (10)	0.0682 (12)	0.0054 (9)	−0.0037 (10)	−0.0101 (8)
C6	0.0618 (11)	0.0374 (8)	0.0703 (12)	0.0011 (8)	0.0095 (9)	−0.0023 (8)
C7	0.0437 (9)	0.0356 (8)	0.0567 (9)	0.0099 (7)	0.0073 (7)	0.0093 (7)
C8	0.0596 (11)	0.0477 (9)	0.0584 (10)	0.0104 (8)	0.0066 (8)	0.0126 (8)
C9	0.0797 (14)	0.0735 (14)	0.0735 (13)	−0.0002 (11)	0.0189 (11)	0.0176 (11)
C10	0.0970 (17)	0.0832 (15)	0.0568 (12)	0.0346 (13)	−0.0021 (11)	0.0040 (10)
Cl2	0.1111 (5)	0.0687 (3)	0.0604 (3)	0.0094 (3)	0.0316 (3)	0.0223 (2)
O2	0.0880 (10)	0.0318 (6)	0.0763 (8)	0.0136 (6)	0.0275 (7)	0.0190 (6)
N2	0.0518 (8)	0.0259 (6)	0.0483 (7)	0.0061 (5)	0.0106 (6)	0.0073 (5)
C11	0.0379 (8)	0.0299 (7)	0.0447 (8)	0.0025 (6)	0.0036 (6)	0.0037 (6)
C12	0.0499 (9)	0.0299 (7)	0.0508 (9)	0.0097 (6)	0.0084 (7)	0.0073 (6)
C13	0.0516 (9)	0.0404 (8)	0.0446 (8)	0.0056 (7)	0.0048 (7)	0.0046 (7)
C14	0.0440 (9)	0.0428 (8)	0.0485 (9)	0.0040 (7)	0.0046 (7)	−0.0027 (7)
C15	0.0528 (10)	0.0358 (8)	0.0636 (10)	0.0134 (7)	0.0093 (8)	0.0003 (7)
C16	0.0519 (9)	0.0322 (7)	0.0581 (10)	0.0102 (7)	0.0080 (8)	0.0101 (7)
C17	0.0458 (9)	0.0334 (8)	0.0480 (9)	0.0021 (6)	0.0059 (7)	0.0091 (6)
C18	0.0618 (11)	0.0465 (9)	0.0510 (9)	0.0088 (8)	0.0151 (8)	0.0134 (7)
C19	0.0980 (16)	0.0619 (11)	0.0592 (11)	0.0223 (11)	0.0262 (11)	0.0215 (9)
C20	0.0684 (12)	0.0652 (12)	0.0567 (11)	0.0089 (10)	0.0157 (9)	−0.0078 (9)

### Geometric parameters (Å, °)

**Table:**

Cl1—C8	1.7731 (19)	Cl2—C18	1.7539 (16)
O1—C7	1.2213 (18)	O2—C17	1.2117 (18)
N1—C7	1.343 (2)	N2—C17	1.3454 (19)
N1—C1	1.426 (2)	N2—C11	1.4171 (19)
N1—H1N	0.8600	N2—H2N	0.8600
C1—C6	1.382 (2)	C11—C12	1.385 (2)
C1—C2	1.385 (2)	C11—C16	1.388 (2)
C2—C3	1.391 (2)	C12—C13	1.385 (2)
C2—H2	0.9300	C12—H12	0.9300
C3—C4	1.395 (2)	C13—C14	1.398 (2)
C3—C9	1.511 (3)	C13—C19	1.503 (2)
C4—C5	1.387 (3)	C14—C15	1.383 (2)
C4—C10	1.514 (3)	C14—C20	1.511 (2)
C5—C6	1.367 (3)	C15—C16	1.376 (2)
C5—H5	0.9300	C15—H15	0.9300
C6—H6	0.9300	C16—H16	0.9300
C7—C8	1.516 (2)	C17—C18	1.517 (2)
C8—H8A	0.9700	C18—H18A	0.9700
C8—H8B	0.9700	C18—H18B	0.9700
C9—H9A	0.9600	C19—H19A	0.9600
C9—H9B	0.9600	C19—H19B	0.9600
C9—H9C	0.9600	C19—H19C	0.9600
C10—H10A	0.9600	C20—H20A	0.9600
C10—H10B	0.9600	C20—H20B	0.9600
C10—H10C	0.9600	C20—H20C	0.9600
			
C7—N1—C1	127.45 (13)	C17—N2—C11	128.24 (12)
C7—N1—H1N	116.3	C17—N2—H2N	115.9
C1—N1—H1N	116.3	C11—N2—H2N	115.9
C6—C1—C2	118.88 (16)	C12—C11—C16	118.87 (14)
C6—C1—N1	118.09 (15)	C12—C11—N2	116.84 (13)
C2—C1—N1	122.98 (14)	C16—C11—N2	124.27 (13)
C1—C2—C3	120.99 (15)	C11—C12—C13	121.92 (14)
C1—C2—H2	119.5	C11—C12—H12	119.0
C3—C2—H2	119.5	C13—C12—H12	119.0
C2—C3—C4	119.72 (16)	C12—C13—C14	119.13 (15)
C2—C3—C9	119.26 (16)	C12—C13—C19	119.77 (15)
C4—C3—C9	121.02 (17)	C14—C13—C19	121.09 (16)
C5—C4—C3	118.29 (16)	C15—C14—C13	118.25 (15)
C5—C4—C10	120.04 (17)	C15—C14—C20	120.87 (16)
C3—C4—C10	121.68 (18)	C13—C14—C20	120.88 (16)
C6—C5—C4	121.78 (16)	C16—C15—C14	122.68 (15)
C6—C5—H5	119.1	C16—C15—H15	118.7
C4—C5—H5	119.1	C14—C15—H15	118.7
C5—C6—C1	120.34 (17)	C15—C16—C11	119.14 (14)
C5—C6—H6	119.8	C15—C16—H16	120.4
C1—C6—H6	119.8	C11—C16—H16	120.4
O1—C7—N1	124.51 (15)	O2—C17—N2	124.59 (15)
O1—C7—C8	121.48 (15)	O2—C17—C18	123.45 (14)
N1—C7—C8	114.00 (14)	N2—C17—C18	111.93 (13)
C7—C8—Cl1	109.74 (12)	C17—C18—Cl2	112.79 (12)
C7—C8—H8A	109.7	C17—C18—H18A	109.0
Cl1—C8—H8A	109.7	Cl2—C18—H18A	109.0
C7—C8—H8B	109.7	C17—C18—H18B	109.0
Cl1—C8—H8B	109.7	Cl2—C18—H18B	109.0
H8A—C8—H8B	108.2	H18A—C18—H18B	107.8
C3—C9—H9A	109.5	C13—C19—H19A	109.5
C3—C9—H9B	109.5	C13—C19—H19B	109.5
H9A—C9—H9B	109.5	H19A—C19—H19B	109.5
C3—C9—H9C	109.5	C13—C19—H19C	109.5
H9A—C9—H9C	109.5	H19A—C19—H19C	109.5
H9B—C9—H9C	109.5	H19B—C19—H19C	109.5
C4—C10—H10A	109.5	C14—C20—H20A	109.5
C4—C10—H10B	109.5	C14—C20—H20B	109.5
H10A—C10—H10B	109.5	H20A—C20—H20B	109.5
C4—C10—H10C	109.5	C14—C20—H20C	109.5
H10A—C10—H10C	109.5	H20A—C20—H20C	109.5
H10B—C10—H10C	109.5	H20B—C20—H20C	109.5
			
C7—N1—C1—C6	−140.52 (16)	C17—N2—C11—C12	−164.70 (14)
C7—N1—C1—C2	42.3 (2)	C17—N2—C11—C16	16.8 (2)
C6—C1—C2—C3	1.1 (2)	C16—C11—C12—C13	−1.1 (2)
N1—C1—C2—C3	178.30 (14)	N2—C11—C12—C13	−179.64 (14)
C1—C2—C3—C4	−0.8 (2)	C11—C12—C13—C14	0.1 (2)
C1—C2—C3—C9	180.00 (16)	C11—C12—C13—C19	−178.89 (16)
C2—C3—C4—C5	0.2 (2)	C12—C13—C14—C15	0.7 (2)
C9—C3—C4—C5	179.41 (17)	C19—C13—C14—C15	179.67 (16)
C2—C3—C4—C10	−179.69 (17)	C12—C13—C14—C20	−178.87 (15)
C9—C3—C4—C10	−0.5 (3)	C19—C13—C14—C20	0.1 (3)
C3—C4—C5—C6	0.0 (3)	C13—C14—C15—C16	−0.5 (3)
C10—C4—C5—C6	179.91 (18)	C20—C14—C15—C16	179.05 (16)
C4—C5—C6—C1	0.4 (3)	C14—C15—C16—C11	−0.5 (3)
C2—C1—C6—C5	−0.9 (3)	C12—C11—C16—C15	1.2 (2)
N1—C1—C6—C5	−178.21 (16)	N2—C11—C16—C15	179.71 (14)
C1—N1—C7—O1	−2.7 (3)	C11—N2—C17—O2	−3.0 (3)
C1—N1—C7—C8	178.29 (15)	C11—N2—C17—C18	175.03 (14)
O1—C7—C8—Cl1	73.33 (19)	O2—C17—C18—Cl2	−15.1 (2)
N1—C7—C8—Cl1	−107.59 (15)	N2—C17—C18—Cl2	166.82 (11)

### Hydrogen-bond geometry (Å, °)

**Table:**

*D*—H···*A*	*D*—H	H···*A*	*D*···*A*	*D*—H···*A*
N1—H1N···O2	0.86	2.05	2.8992 (16)	172
N2—H2N···O1^i^	0.86	2.14	2.9802 (16)	166

Symmetry codes: (i) *x*, *y*−1, *z*.
